# Genome-wide association study reveals a *GLYCOGEN SYNTHASE KINASE 3* gene regulating plant height in *Brassica napus*

**DOI:** 10.3389/fpls.2022.1061196

**Published:** 2022-11-02

**Authors:** Chuanji Zhao, Li Yang, Minqiang Tang, Lijiang Liu, Junyan Huang, Chaobo Tong, Yang Xiang, Shengyi Liu, Xiaohui Cheng, Meili Xie

**Affiliations:** ^1^ Key Laboratory of Biology and Genetic Improvement of Oil Crops, The Ministry of Agriculture and Rural Affairs, Oil Crops Research Institute, Chinese Academy of Agricultural Sciences, Wuhan, Hubei, China; ^2^ Biosystematics Group, Wageningen University and Research, Wageningen, Netherlands; ^3^ Key Laboratory of Genetics and Germplasm Innovation of Tropical Special Forest Trees and Ornamental Plants (Ministry of Education), School of Forestry, Hainan University, Haikou, China; ^4^ Guizhou Rapeseed Institute, Guizhou Academy of Agricultural Sciences, Guiyang, Guizhou, China

**Keywords:** plant height, genome-wide association study (GWAS), rapeseed (*B. napus* L.), RNA sequencing (RNA-Seq), *GSK3* gene family

## Abstract

Rapeseed (*Brassica napus*) is an allotetraploid crop that is the main source of edible oils and feed proteins in the world. The ideal plant architecture breeding is a major objective of rapeseed breeding and determining the appropriate plant height is a key element of the ideal plant architecture. Therefore, this study aims to improve the understanding of the genetic controls underlying plant height. The plant heights of 230 rapeseed accessions collected worldwide were investigated in field experiments over two consecutive years in Wuhan, China. Whole-genome resequencing of these accessions yielded a total of 1,707,194 informative single nucleotide polymorphisms (SNPs) that were used for genome-wide association analysis (GWAS). GWAS and haplotype analysis showed that *BnaA01g09530D*, which encodes BRASSINOSTEROID-INSENSITIVE 2 and belongs to the *GLYCOGEN SYNTHASE KINASE 3* (*GSK3*) family, was significantly associated with plant height in *B. napus*. Moreover, a total of 31 *BnGSK3s* with complete domains were identified from *B. napus* genome and clustered into four groups according to phylogenetic analysis, gene structure, and motif distribution. The expression patterns showed that *BnGSK3s* exhibited significant differences in 13 developmental tissues in *B. napus*, suggesting that *BnGSK3s* may be involved in tissue-specific development. Sixteen *BnGSK3* genes were highly expressed the in shoot apical meristem, which may be related to plant height or architecture development. These results are important for providing new haplotypes of plant height in *B. napus* and for extending valuable genetic information for rapeseed genetic improvement of plant architecture.

## Introduction

Rapeseed (*Brassica napus* L., 2n = 38, AACC) is the main source of edible oils and feed proteins worldwide. However, the rapeseed industry is currently confronted with multiple bottlenecks, i.e. low yield, low planting density, low mechanization degree, large amount of fertilization, and high labor costs, which seriously impacts the sustainable development of the rapeseed industry. Shaping the ideal plant architecture of rapeseed is helpful to break through these bottlenecks, but the lack of a clear genetic basis and constituent elements has hindered the development of this research. Plant height is one of the most important determinants of ideal plant architecture. Since lodging is a common phenomenon and yield loss caused by lodging is severe (16.2%) in rapeseed production (Islam and Evans, 1994). Therefore, moderate dwarfing of crop plant height increased the harvest index.

Plant height is an agronomic trait with complex genetic basis. It is easily affected by environment and usually regulated by both major and minor genes. In recent years, with the rise of the green revolution in wheat, breeders have identified a large number of quantitative trait loci (QTLs) controlling wheat plant height on 21 chromosomes using different populations and markers (Chu et al., 2008; Buerstmayr et al., 2011; Guo et al., 2018). The green revolution in rice began with the application of a semi-dwarf gene *sd1* (Monna et al., 2002; Sasaki et al., 2002; Spielmeyer et al., 2002). The discovery and utilization of dwarf mutants and corresponding genes have greatly promoted the development of new rice varieties. The cloned dwarf genes in rice are mainly involved in the biosynthesis and signal pathways of plant hormones (e.g., gibberellin, brassinolide, and strigolactone). Some of these genes contain special domains, including *sd1* (Ye et al., 2015), *D1* (Ferrero-Serrano et al., 2018; Sun et al., 2018), *GID1* and *GID2* (Hirano et al., 2010), *OsDWARF4* (Fang et al., 2016), and *OsTB1* (Fang et al., 2020). Currently, the only known gene responsible for ideal plant architecture gene in rice is *IPA1*, which encodes the squamosa-like promoter-binding protein OsSPL14. Mutations in *OsSPL14* reduced tillering, increased grain number per ear and 1000-grain weight, thickened stem, and enhanced lodging resistance, thereby increasing the yield (Jiao et al., 2010; Miura et al., 2010).

In *B. napus*, the identification of QTLs highly related with plant height is an important task in genetic maps and genome-wide association analysis (GWAS). Fourteen QTLs for plant height were identified in different linkage groups using a recombinant inbred line (Cai et al., 2014). A major plant height QTL on chromosome A10, was identified by whole-genome resequencing (WGS) based genetic mapping (Dong et al., 2021). Using the Illumina Brassica 60 K Bead Chip Array and a diversity of 520 accessions, a total of 68 plant height-related loci were obtained by GWAS under six environments. Most of the genes in these loci were involved in gibberellin synthesis and signal pathway (Sun et al., 2016). In recent years, progress has been made in the exploitation of dwarf genetic resources and genes in *B. napus*. Most dwarf mutants belong to gibberellin, auxin, and brassinolide-insensitive mutants. In two *B. napus* dwarf mutants of approximately 70 cm height, their candidate genes were mapped on chromosomes A06 and C07, both of which encode DELLA proteins, a negative regulator of the gibberellin signal transduction pathway, and have missense mutations in the VHYNP domain (Liu et al., 2010; Zhao et al., 2017). Mutations at different sites of *BnaC05g29300D*, encoding an auxin signaling transport repressor, resulted in rapeseed plant heights of only 25 cm (Zhao et al., 2019; Zheng et al., 2019). Mutation of *BnaA3.IAA7*, which encodes an auxin-inducible protein, disrupted the conserved degradation motif GWPPV and reduced the affinity between BnaA3.IAA7 and the transport inhibitor in an auxin dose-dependent manner, thus inhibiting BnaA3.IAA7 degradation and auxin signaling in *B. napus* dwarf mutant *sca* (Li et al., 2019a). The dwarf locus *BnDWARF2* was mapped to a 34.62 kb interval, in which *BnaC04g41660D* encoding a GLYCOGEN SYNTHASE KINASE 3 (GSK3-like) in the brassinosteroid signaling, was the causal gene controlling plant height in oilseed rape (Yang et al., 2021). In addition, other genes unrelated to plant hormones may also be involved in the regulation of plant height in *B. napus*; for example, the Octicosapeptide/Phox/Bem1p family protein encoding gene *BnaC09g20450D* contains a single nucleotide polymorphism (SNP) that co-segregates with the dwarf phenotype in *df59* mutant (Wang et al., 2020a).

Although many plant height QTLs and dwarf genes have been identified, they have not been fully utilized in breeding, and cultivars with dwarf or semi-dwarf phenotypes are still the major objective in rapeseed breeding. This study aims to better understand the genetic control of plant height and to unearth more valuable information from the genome of polyploid rapeseed based on GWAS for plant height in 230 core rapeseed accessions around the world. We identified *BnaA01g09530D*, a *BnGSK3* gene involved in the cross-talk between auxin and brassinosteroid signaling pathways, was significantly associated with plant height. We also analyzed the expression pattern in various tissues, overall distribution in the rapeseed genome, and phylogenetic analysis of the *BnGSK3s* family.

## Results

### Phenotype variation of plant height in 230 *B. napus* accessions

Extensive phenotypic variations of plant height were observed in 230 inbred accessions over two consecutive years (**Table 1**). The plant height ranged from 149.23–230.59 cm in 2017–2018 and from 115.12–189.28 cm in 2018–2019, suggesting that the environment factors had a great impact on plant height (**Supplementary Figure 1A** and **Table 1**). The plant heights in 2017–2018, 2018–2019, and the BLUP of the 230 rapeseed accessions displayed normal distributions (**Supplementary Figure 1A**). The coefficient of variation in 2018–2019 was 9.10%, which was higher than that in 2017–2018 (7.38%) and BLUP (7.69%) (**Table 1**). Nevertheless, no significant difference was observed between the phenotype of 2017-2018 and 2018-2019, as shown by the correlation analysis (R^2^ > 0.70) (**Supplementary Figure 1B**). These analyses revealed that the phenotype of 230 rapeseed accessions were reliable and feasible for association analysis.

**Table 1:** Phenotypic variations of plant height in rapeseed natural population.

Environment	Min	Max	Mean	SE	SD	Var	Kurtosis	Skewness	CV (%)
2017–2018	149.23	230.59	190.88	0.93	14.08	198.33	0.051	0.055	7.38
2018–2019	115.12	189.28	150.07	0.9	13.66	186.67	-0.039	0.209	9.1
BLUP	132.75	206.85	170.25	0.86	13.09	171.28	0.138	0.098	7.69

Min, minimum value; Max, maximum value; Mean, mean value; SE, standard error; SD, standard deviation; Var, variance; CV, coefficient of variation; BLUP, best linear unbiased prediction.

### Genomic variation of rapeseed resequencing population

A total of 230 rapeseed accessions, consisting of 25 spring-, 33 winter-, and 172 semi-winter ecotypes, were employed for WGS (**Supplementary Table 1**). Approximately 1,097.37 Gb data were generated, with an average size of 4.77 Gb and an average depth of 6.46 × depth per accession (**Supplementary Table 1**). The average coverage of *B. napus* reference genome was 82.19% (**Supplementary Table 1**). A total of 1,707,194 informative SNPs were acquired with an average of 94,844 SNPs on each chromosome (**Table 2**). The density of SNPs on different chromosomes ranged from 1.01 to 4.86 SNP/kb, with chromosome C09 having the lowest density and A10 the highest (**Table 2**). These results suggested the reliability of SNP information and could be used for further analyses.

**Table 2:** Statistics of SNP number and density on each chromosome.

Chromosome	Length	SNPs	SNP/kb
chrA01	23,267,856	80,834	3.47
chrA02	24,793,737	75,701	3.05
chrA03	29,767,490	122,543	4.12
chrA04	19,151,660	83,705	4.37
chrA05	23,067,598	104,826	4.54
chrA06	24,396,386	117,969	4.84
chrA07	24,006,521	114,454	4.77
chrA08	18,961,941	68,741	3.63
chrA09	33,865,340	126,210	3.73
chrA10	17,398,227	84,541	4.86
chrC01	38,829,317	109,044	2.81
chrC02	46,221,804	82,943	1.79
chrC03	60,573,394	129,746	2.14
chrC04	48,930,237	120,199	2.46
chrC05	43,185,227	53,918	1.25
chrC06	37,225,952	74,092	1.99
chrC07	44,770,477	78,036	1.74
chrC08	38,477,087	79,692	2.07
chrC09	48,508,220	49,213	1.01

### Identification of *BnGSK3* significantly associated with plant height

**Figure 1:**
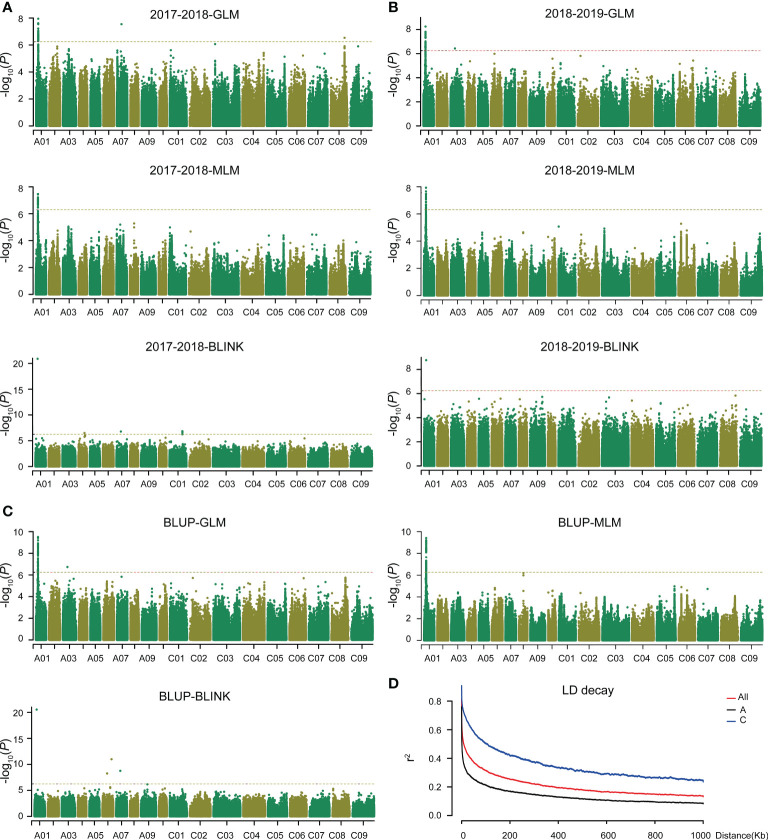
GWAS of plant height in *Brassica napus* and LD decay analysis. The threshold value is -log_10_(1/SNPs number). **(A)** GWAS of plant height in 2017–2018 based on GLM, MLM, and BILNK models. **(B)** GWAS of plant height in 2018–2019 based on GLM, MLM, and BILNK models. **(C)** GWAS of plant height for BLUP based on GLM, MLM, and BLINK models. **(D)** Linkage disequilibrium (LD) decay of A and C sub-genomes and whole genome.

According to the Q+K model, the associated population could be divided into nine subgroups (**Supplementary Figure 2A, B**). More than 90% of the relative kinship coefficients among these accessions were found to be lower than 0.1, suggesting that most accessions in this population lacked or had weak genetic relatedness (**Supplementary Figure 2C**). The average linkage disequilibrium (LD) decay of the A and C sub-genomes were 4.1 and 120.3 kb, respectively. It was 33.4 kb for the whole genome (A + C) when r^2^ decayed to its half (**Figure 1D**).

**Figure 2:**
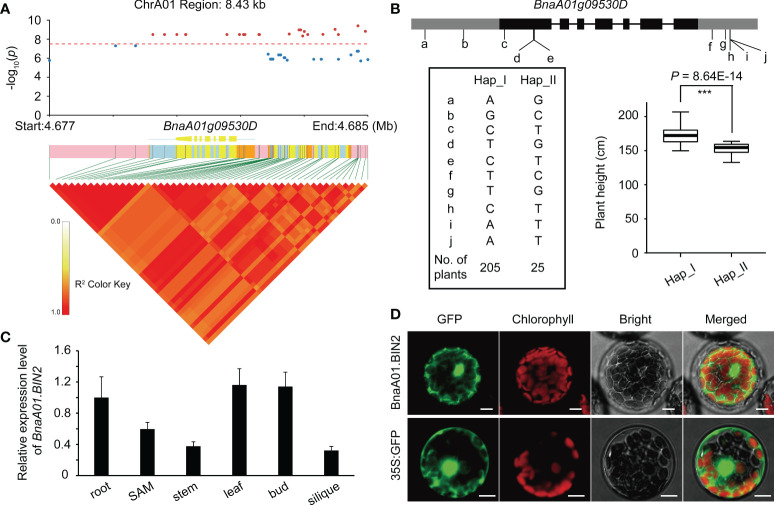
Integrated characteristic of *BnA01.BIN2*. **(A)** LD block analysis of significant SNPs in BLUP based on MLM models. The threshold value is -log_10_(0.05/SNPs number). **(B)** Gene structure of *BnaA01.BIN2* and haplotype analysis. **(C)** Subcellular localization of *BnaA01.BIN2* in *Arabidopsis* protoplasts. Green fluorescence, GFP; red fluorescence, chloroplast autofluorescence; Merged, merged images of all channels. Bar = 10 μm. **(D)** Expression pattern of *BnaA01.BIN2*.

To dissect the genetic control of plant height in *B. napus*, we performed GWAS in two consecutive years. A significant locus on chromosome A01 was simultaneously identified using GLM, MLM, and BLINK models (**Figures 1A–C**, and **Supplementary Table 3**). Quantile-quantile plots showed obvious deviations between the observed and expected values, indicating the selected models were correct and suitable for GWAS (**Supplementary Figure 3**). Within the significance interval, 806 SNPs were repeatedly identified in different environments and models (GLM and MLM) (**Supplementary Table 3**). According to the MLM model in BLUP and LD decay of A sub-genome (**Figure 1**), three genes (*BnaA01g09530D*, *BnaA01g09540D*, and *BnaA01g09550D*) near the significant SNPs were strongly associated (**Figure 2A**). Based on the annotation of the *B. napus* reference genome, *BnaA01g09530D*, encoding BRASSINOSTEROID-INSENSITIVE 2 (BIN2) and involving in the brassinosteroid signaling pathway, may be a candidate gene controlling plant height in *B. napus*. In addition, the position of co-identified SNP by BLINK model in different environments was 4,772,232 (**Supplementary Table 3**), which was far away from the co-identified significant SNPs in GLM and MLM, due to the different algorithm principle of BLINK. In the application of BLINK, a bin, containing all the linked SNPs in a region, is taken as a unit, rather than a single SNP as a unit like GLM and MLM (Huang et al., 2019), suggesting that *BnaA01g09530D* was also identified in the BLINK models. A total of ten SNPs variations were observed in the sequence of *BnaA01.BIN2*. Haplotype analysis of these ten SNPs revealed favorable allelic variation (Hap_II), conferring a significant reduction in plant height (**Figure 2B**).

The expression pattern of *BnaA01.BIN2* showed that it was highly expressed in leaves, buds, and roots, followed by SAM, suggesting that it plays an important role in plant development. Subcellular localization, as indicated by green fluorescent protein (GFP), showed that BnaA01.BIN2 was localized in nucleus and cytoplasm (**Figure 2D**).

### *In silico* analysis of *BnGSK3s* in rapeseed genome

Candidate gene *BnaA01.BIN2* belongs to the *glycogen synthase kinase 3* (GSK3) gene family. Using protein sequences of AtGSK3s as the query of BLAST, a total of 38 *BnGSK3s* were identified in “*Darmor-bzh*” rapeseed genome, and 31 *BnGSK3s* with Pkinase domain were finally extracted (**Table 3**). Of these *BnGSK3s*, 16% (5) resulted from dispersed duplications and 84% (26) originated from whole-genome duplication (WGD) or segmental duplication (**Table 3**). Most *AtGSK3s* have several syntenic genes in *B. napus*, among which *BnBIN2* contains six homologous genes and is the largest member of *BnGSK3s* (**Supplementary Table 2**). However, there were no homologous genes for *AtBIL2* and *AtSK42* in *B. napus* (**Supplementary Table 2**). We identified 15 *BrGSK3s* and 16 *BoGSK3s* according to the *Brassica* Database (BRAD) (http://brassicadb.cn/) and no homologous genes of *AtBIL2* and *AtSK42* were identified in the reference genomes of *B. rapa* (Brara_Chiifu_V3.5) and *B. oleracea* (Braol_JZS_V2.0) (**Supplementary Table 2**). These results suggested that the *GSK3s* family is highly conserved in *Brassicaceae*, whereas the loss of *BIL2s* and *SK42s* may occur prior to *Brassicaceae* speciation.

**Table 3:** The information of *BnGSK3s* family in rapeseed.

Gene ID	Chromosome	AAs	pI	MW (kDa)	Duplication type
*BnaAnng02930D*	Ann_random	405	6.38	46.08	WGD or Segmental
*BnaA09g38810D*	A09	438	7.61	49.67	WGD or Segmental
*BnaAnng35300D*	Ann_random	375	8.85	42.45	Dispersed
*BnaA09g04100D*	A09	407	8.7	46.21	WGD or Segmental
*BnaA05g31460D*	A05	515	8.97	58.72	WGD or Segmental
*BnaC07g50210D*	C07_random	375	8.74	42.43	WGD or Segmental
*BnaA08g26100D*	A08	422	8.37	47.67	WGD or Segmental
*BnaCnng48480D*	Cnn_random	422	8.37	47.66	Dispersed
*BnaA05g11700D*	A05	225	7.57	25.35	WGD or Segmental
*BnaC03g62810D*	C03	381	8.58	43.08	WGD or Segmental
*BnaCnng52760D*	Cnn_random	375	8.85	42.42	Dispersed
*BnaCnng13510D*	Cnn_random	433	6.87	49.3	WGD or Segmental
*BnaAnng31110D*	Ann_random	341	8.72	38.99	Dispersed
*BnaC03g34380D*	C03	412	8.56	46.8	WGD or Segmental
*BnaA09g51790D*	A09_random	479	7.92	53.5	WGD or Segmental
*BnaA07g18960D*	A07	433	7.2	49.35	WGD or Segmental
*BnaC05g07320D*	C05	418	8.39	47.43	WGD or Segmental
*BnaC04g41660D*	C04	411	8.74	46.28	WGD or Segmental
*BnaC01g11150D*	C01	382	8.44	43.11	WGD or Segmental
*BnaA03g29180D*	A03	412	8.56	46.8	WGD or Segmental
*BnaA03g27010D*	A03	469	6.71	52.69	WGD or Segmental
*BnaC05g46010D*	C05	411	8.52	46.65	WGD or Segmental
*BnaCnng02170D*	Cnn_random	472	8.2	52.69	WGD or Segmental
*BnaA03g05700D*	A03	569	8.89	62.99	Dispersed
*BnaA01g09530D*	A01	375	8.7	42.41	WGD or Segmental
*BnaC07g28590D*	C07	403	8.7	45.85	WGD or Segmental
*BnaC03g06580D*	C03	410	8.65	46.03	WGD or Segmental
*BnaC09g03480D*	C09	407	8.7	46.18	WGD or Segmental
*BnaA06g28290D*	A06	404	8.59	45.83	WGD or Segmental
*BnaC03g31970D*	C03	467	6.89	52.36	WGD or Segmental
*BnaA06g05770D*	A06	418	8.39	47.43	WGD or Segmental

AAs, amino acids; pI, isoelectric point; MW, molecular weight; WGD, whole-genome duplication; random, contigs unassembled on chromosomes.

**Figure 3:**
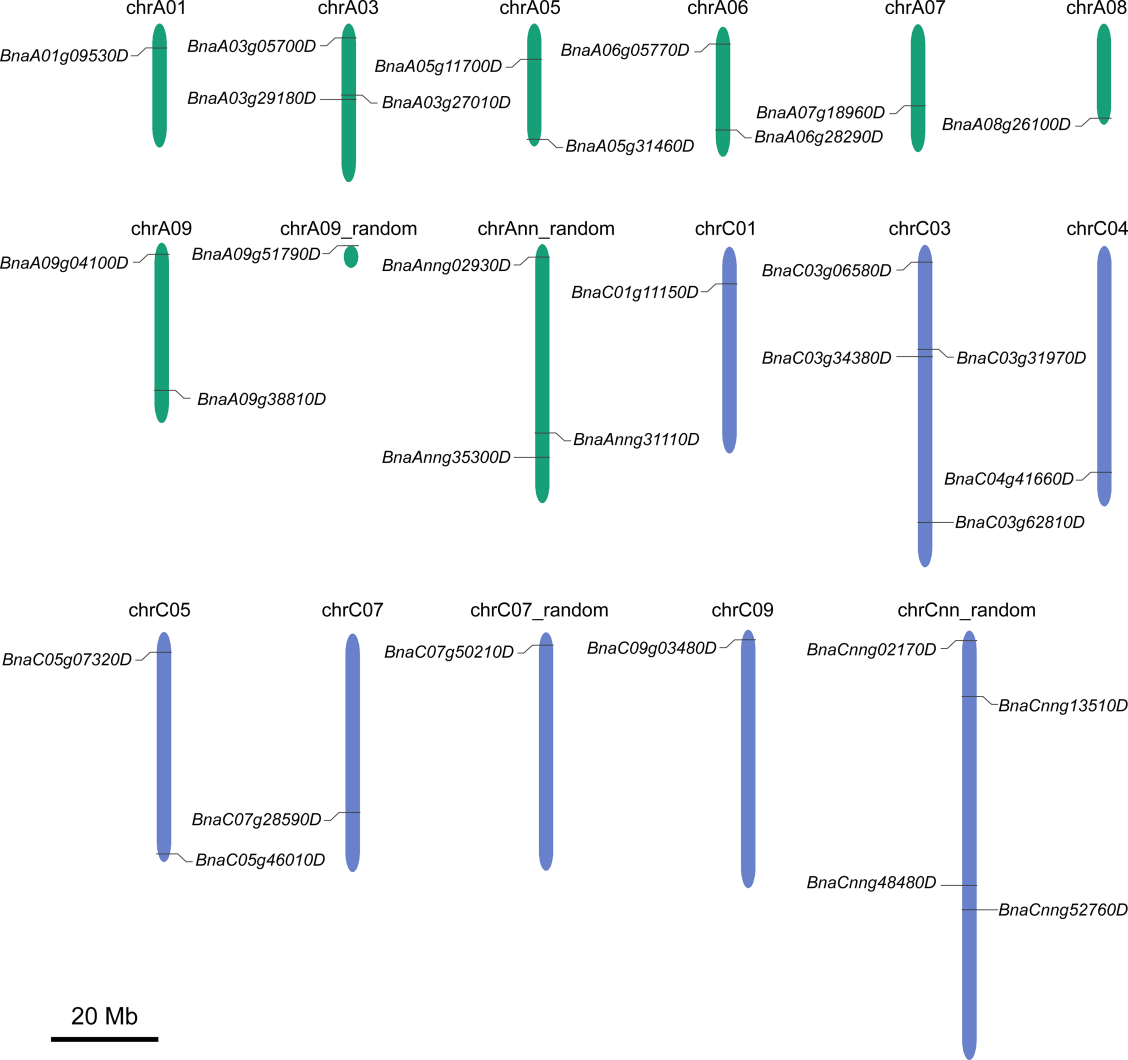
Chromosomal distribution of *BnGSK3s* in the genome of *Brassica Napus*. Green and blue chromosomes represent A and C sub-genome of *B*. *napus*, respectively.

The 31 *BnGSK3s* were unevenly distributed in 13 chromosomes and four random chromosomes, 16 and 15 *BnGSK3s* were located on the A and C sub-genomes, respectively (**Figure 3** and **Table 3**).

### Phylogenetic, syntenic relationship, and conservation analysis of *BnGSK3s*

**Figure 4:**
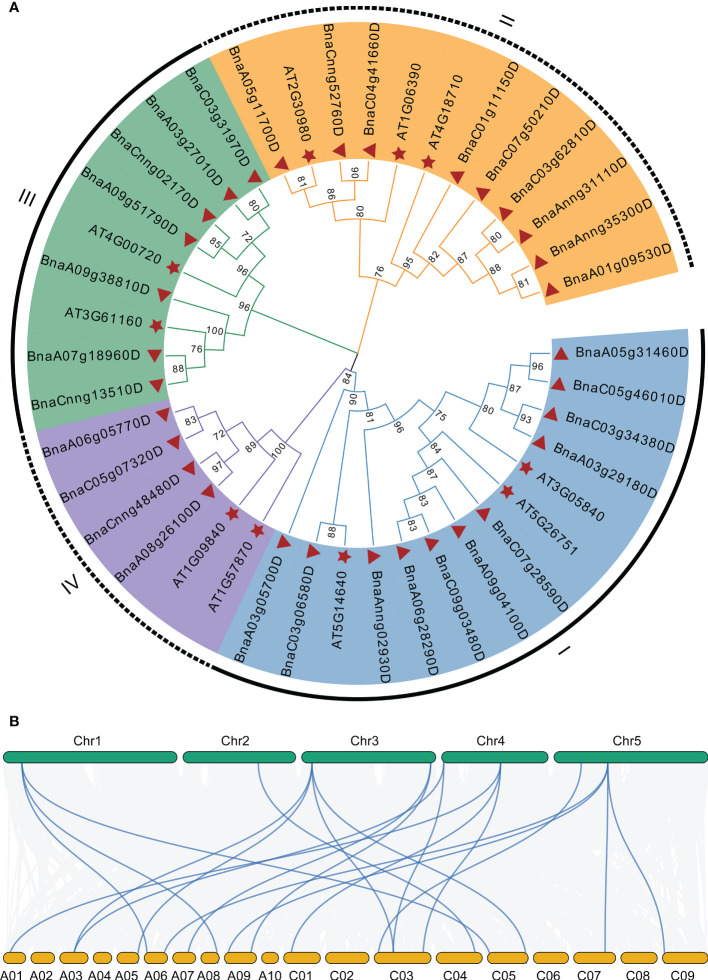
Phylogenetic and syntenic analysis of *AtGSK3s* and *BnGSK3s*. **(A)** Phylogenetic analysis. **(B)** syntenic analysis. The green and yellow blocks represent *Arabidopsis* and *B*. *napus* chromosome, respectively.

To explore the phylogenetic relationship of *GSK3s* family, we constructed a phylogenetic tree using GSK3s protein sequences from *Arabidopsis* and *B. napus*. The 10 *AtGSK3s* and 31 *BnGSK3s* were divided into four groups: Group I (SK11, SK12, and SK13), Group II (BIN2, BIL1, and BIL2), Group III (SK31 and SK32), and Group IV (SK41 and SK42) (**Figure 4A** and **Supplementary Table 2**). Group I had 22 *GSK3s*, including 11 *BnGSK3s*, 5 *BrGSK3s*, and 6 *BoGSK3s*, accounting for the largest group. Group IV was the smallest, with only eight *GSK3s* (**Figure 4A** and **Supplementary Table 2**). This suggests that Group I of *GSK3s* was more expanded compared to that of Group IV. Within each group, *BnGSK3s* belonging to the A and C sub-genomes in *B. napus*, along with the *AtGSK3s* in *Arabidopsis*, clustered into a small clade (**Figure 4A**), suggesting that the phylogenetic relationship of GSK3 was consistent with the evolution of rapeseed. The syntenic analysis between *AtGSK3s* and *BnGSK3s* showed that most of *AtGSK3s* have over two syntenic genes in B. napus (**Figure 4B**), which is consistent with phylogenetic relationship of *GSK3s*.

**Figure 5:**
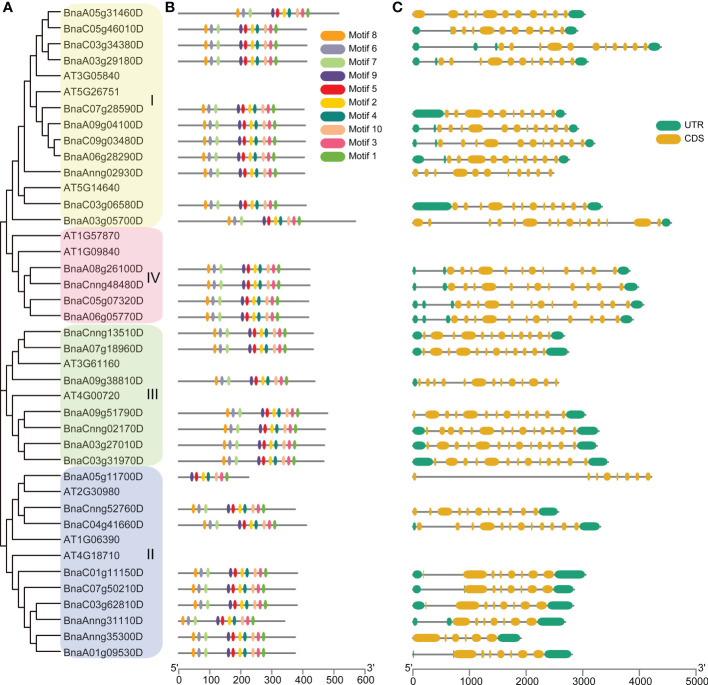
Gene structure and conserved motif analyses of *AtGSK3s* and *BnGSK3s*. **(A)** Phylogenetic tree of *AtGSK3s* and *BnGSK3s*. **(B)** Conserved motifs of *AtGSK3s* and *BnGSK3s*. **(C)** Gene structures of *AtGSK3s* and *BnGSK3s*.

To explore the conservation of *BnGSK3s*, gene structure and protein motifs were analyzed (**Figure 5**). In general, gene structures of the 31 *BnGSK3s* differed obviously between different groups. Among them, the syntenic genes showed relatively similar gene structures (**Figures 5A, C**). The gene structures of approximately 74% of *BnGSK3s* (23) exhibited 5’ and 3’ untranslated regions (UTR) (**Figure 5C**). Six *BnGSK3s* (*BnA05g31460D*, *BnA03g05700D*, *BnA09g38810D*, *BnA09g51790D*, *BnCnng52760D*, *and BnAnng35300D*) only had 5-’ or 3-UTR (**Figure 5C**). In addition, all *BnGSK3s* contained exons and introns (**Figure 5C**). As for motif analysis, except *BnaA05g11700D* possessed seven conserved motifs, the remaining *BnGSK3s* had ten conserved motifs (**Figure 5B**). These results suggested that the core sequences of the *BnGSK3s* were conserved.

### Expression patterns of *BnGSK3s*

**Figure 6:**
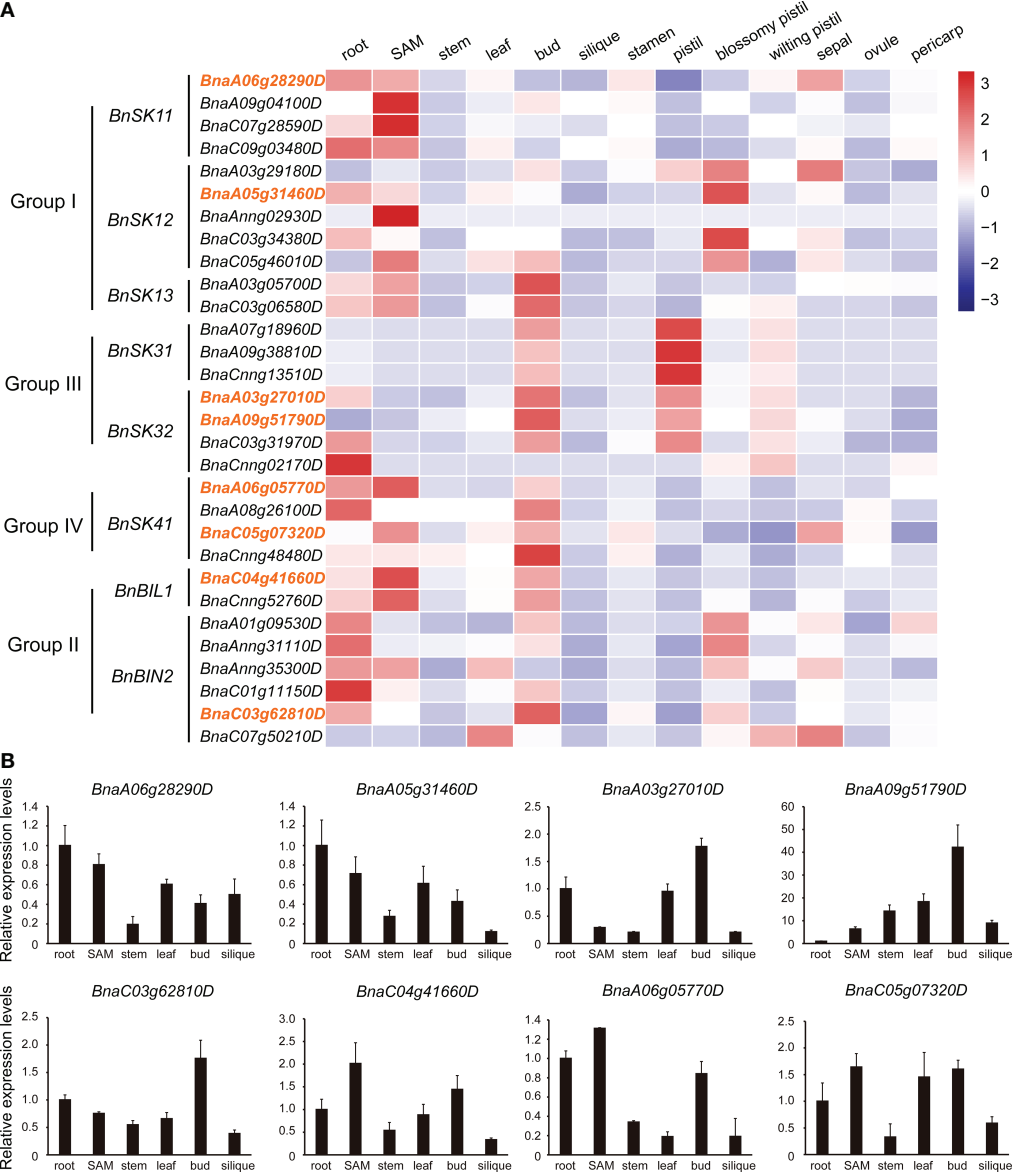
Expression pattern of *BnGSK3s*. **(A)** Expression pattern of the 31 *BnGSK3s* in 13 tissues of *B*. *napus* cv. ZS11 based on RNA-seq. Bar represents the normalized transformed counts of FPKM. The expression of eight genes marked in orange were verified by qRT-PCR.**(B)** Expression patterns of eight *BnGSK3s* in six tissues of *B*. *napus* cv. ZS11 based on qRT-PCR.

Based on published transcriptome data (Li et al., 2019b; Dong et al., 2021), the expression patterns of the 31 *BnGSK3s* in 13 tissues of ZS11 were analyzed, which showed that the *BnGSK3s* were expressed in different tissues (**Figure 6A**). However, a set of homologous genes, including *BnSK31*, *BnBIL1*, and *BnBIN2*, showed similar expression patterns, suggesting a potential redundancy of function (**Figure 6A**). Different expression patterns were observed within the same group, suggesting functional divergence in *BnGSK3s*. For example, *BnBIL1* was highly expressed in SAM, whereas *BnBIN2* was highly expressed in roots (**Figure 6A**). In addition, *BnSK13s* were prone to express in pistils and buds, suggesting that these genes may be involved in flower development (**Figure 6A**). Thirteen *BnGSK3s* were highly expressed in SAM, indicating that *BnGSK3s* have a certain effect on the development of plant architecture. In addition, we selected eight *BnGSK3s* from different groups to perform qRT-PCR in six tissues, which suggested that the expression pattern was consistent with the RNA-seq data (**Figure 6B**).

## Discussion

### Dilemma of plant architecture breeding and the lack of genetic basis for plant height in rapeseed

Since the “Green Revolution” in the 1960s, researchers have carried out extensive research to come up with ideal plant architecture models for many crops (Teichmann and Muhr, 2015; Liu et al., 2020; Pearce, 2021). Several novel genes controlling aboveground plant architecture have been identified and their regulatory mechanisms have been expounded, laying the foundation for breeding new high-yielding varieties of rice (Monna et al., 2002; Sasaki et al., 2002; Wang and Li, 2008; Wang et al., 2020b), wheat (Chai et al., 2022; Xiong et al., 2022), maize (Phillips et al., 2011; Li et al., 2020a), and soybean (Guo et al., 2020; Chen et al., 2021b). Many researchers have proposed models of ideal rapeseed plant architecture (Liu et al., 2022; Zheng et al., 2022). However, these are only concepts and cannot solve actual problems in production. There are several difficulties in studying the ideal plant architecture of rapeseed: 1) the lack of materials with good plant architecture materials; 2) the uncertainty of proper index used for the research of rapeseed plant architecture; 3) severe environmental impact on plant architecture-related traits; 4) the lack of clear genetic basis. For many crops, such as rice and wheat, plant height has been used as a breakthrough point to study plant architecture (Peng et al., 1999; Hedden, 2003). Therefore, plant height is essential in shaping the ideal plant architecture of crops. Although research progress has been made in the study of plant height traits of rapeseed (Liu et al., 2010; Wang et al., 2016a; Wang et al., 2016b), the genetic basis of rapeseed plant height remains unclear.

Currently, a single genetic resource cannot effectively improve the present plant architecture of rapeseed. GWAS is often used as an effective method to unravel the genetic architecture of complex agronomic traits in crops. Combined with association analysis and linkage analysis, 61 SNPs significantly associated with low zinc tolerance and 15 QTLs were identified in maize. Expression and haplotype analyses were used to mine the favorable allele conferring low zinc tolerance (Xu et al., 2022). Similar study could be found in Guo et al. (2021), in which 63 loci related to stem strength and yield were identified and favorable alleles for both high stem strength and high yield were discovered using 524 rice germplasm resources and 193 recombinant inbred lines (Guo et al., 2021). Based on GWAS and a transcriptome-wide association study, 15 stable QTLs and 1,854 candidate genes were detected in *B. napus*, which were significantly associated with seed glucosinolate content. Haplotype analysis showed that seed low glucosinolate was mainly resulted by the co-action of multiple favorable alleles (Tan et al., 2022). In this study, GWAS was performed on plant height of 230 *B. napus* accessions using three models (GLM, MLM, and BLINK). An unreported gene, *BnaA01.BIN2*, was simultaneously identified by all three models (**Figures 1**, **2**), which increased the confidence of the results. However, no other loci or reported genes were co-identified, probably due to population structure constraints. These results provide insights for subsequent adjustment of population structure to more effectively detect available loci, genes, or favorable alleles.

### *BnBIN2*, a core member of *BnGSK3s*, is involved in plant development and stress response

GSK3 is a group of highly conserved cytoplasmic serine/threonine protein kinases that are widely present in animal and plant cells. These proteins perform their functions mainly by phosphorylating key substrate proteins of different signaling pathways. GSK3 is regulated by a variety of post-translational modification mechanisms. *BnaA01.BIN2*, identified by GWAS in this study (**Figures 1**, **2**), encodes BRASSINOSTEROID-INSENSITIVE 2 (BIN2) and, belongs to the *BnGSK3* family. AtBIN2 plays a role in the crosstalk between auxin and brassinosteroid signaling pathways (https://www.arabidopsis.org/index.jsp). In *B. napus*, *BnaC04.BIL1*, which has been isolated from the dwarf mutant *Bndwarf2* (Yang et al., 2021), encodes BIN2-LIKE 1, and is also a member of *GSK3s*.

As a core member of *GSK3s*, *BIN2* is a constitutively active kinase in plants, whose activity is affected by various regulatory mechanisms, including nucleocytoplasmic distribution, protein-protein interaction strength, phosphorylation and dephosphorylation, acetylation, and ubiquitination (Mao and Li, 2020). The direct function of BIN2 is to participate in the signal transduction pathway of brassinolide, which plays an important role in plant development (Anne et al., 2015). BIN2 directly controls the transcriptional regulatory complex composed of WEREWOLF (WER), transcription factor GLABRA3 (GL3), and WD40 repeat protein TRANSPARENT TESTA GLABRA1 (TTG1), It can phosphorylates GL3 and TTG1 in the WER-GL3-TTG1 complex to inhibit their transcriptional activity, thereby regulating root hair development (Cheng et al., 2014). BIN2 participates in photomorphogenesis by interacting with HY5, an important transcription factor for photomorphogenesis (Li et al., 2020b). In addition, BIN2 is involved in osmotic stress and adverse effects, and can promote lateral root development by phosphorylating auxin-responsive factor ARF7 (Cho et al., 2014). BIN2 is also involved in abscisic acid signal transduction to regulate the osmotic stress response (Wang et al., 2018) and enhances plant drought tolerance by phosphorylating RSPONSIVE OT DESICCATION 26, NAC family transcription factor (Jiang et al., 2019).

*GSK3* is involved in the regulation of plant growth and development. However, only one gene has been reported to be related with plant height in rapeseed (Yang et al., 2021). In this study, to determine the relationship between *BnGSK3s* and plant height in allotetraploid rapeseed, we investigated the *BnGSK3s* family, which consists of 16 homologs in A sub-genome and 15 in C sub-genome (**Figure 3** and **Table 3**). Based on the transcriptome data of 13 tissues in ZS11, the expression pattern of *BnGSK3s* were found to show obvious expression preference difference in rapeseed (**Figure 6**), in which 16 genes were highly expressed in SAM and three were highly expressed in the pistil (**Figure 6A**). Moreover, we identified favorable allelic variations in *BnaA01.BIN2* among 230 *B. napus* accessions, whereas we failed to detect any SNP variation in the corresponding syntenic gene *BnaC01.BIN2* (*BnaC01g11150D*). This could be caused by the limited numbers of accessions used for GWAS in this study. As such, more rapeseed genetic resources should be collected to dissect more favorable allelic variations in *BnGSK3s* for plant height and plant architecture.

## Conclusions

In this study, GWAS was performed on plant heights of a bio-panel of 230 rapeseed accessions in two consecutive years based on three models. The results showed that *BnaA01.BIN2* belonging to *BnGSK3s* family, was significantly associated with plant height in *B. napus*. A total of 31 *BnGSK3s* were identified and clustered into four groups. Expression pattern analysis suggests that *BnGSK3s* may be involved in tissue-specific development. Sixteen *BnGSK3* genes were highly expressed in SAM, which may be related to plant height development. These findings are important for the genetic improvement of plant height and architecture in rapeseed.

## Materials and methods

### Plant materials, growth conditions, and phenotypic analysis

A total of 230 rapeseed cultivars or inbred lines (**Supplementary Table 1**) were collected worldwide, representing the genetic diversity of *B. napus* for GWAS of plant height. Field trials were conducted by a randomized design with three replications. For each accession, 45 individuals were grown in a 2.0 × 1.0 m^2^ plot with three rows in each environment (2017–2018, 2018–2019, winter-spring growing season) in the Yangluo experimental field of Oil Crops Research Institute of the Chinese Academy of Agricultural Sciences, Wuhan, China. The R script lme4 (CRAN-Package lme4 (r-project.org)) and lsmeans were used to calculate the best linear unbiased prediction (BLUP) of each inbred line in the natural population (Zhao et al., 2022).

At the mature stage, 10 plants with good growth and development were randomly selected from each plot for phenotype investigation. The length from the cotyledon node to the apical position of the whole plant was measured and recorded as plant height. The statistics of the phenotype variation and frequency distribution were calculated using SPSS 22 (IBM SPSS, Armonk, NY, United States) (Zhao et al., 2022). The Pearson’s product-moment correlation analysis of plant height between 2017-2018 and 2018-2019 was carried out by R Package.

### Whole-genome sequencing, variant identification and annotation

Total genomic DNA from fresh young leaf tissue of each inbred line (230 accessions) was extracted using a Hi-DNAsecure Plant Kit (TIANGEN, Beijing). DNA libraries were constructed with high-quality genomic DNA and whole-genome resequencing (WGS) was performed using the Illumina NovaSeq 6000 system. Clean data (clean reads) were obtained by filtering the raw data. All clean reads were mapped to the *B. napus* reference genome (*Darmor-bzh* V5, https://www.genoscope.cns.fr/brassicanapus/data/) using the Burrows-Wheeler Aligner software (Li and Durbin, 2009; Chalhoub et al., 2014). SAMTools (parameter: -q 30; http://samtools.sourceforge.net/) and Sentieon Genomics (parameter: –algo Dedup –rmdup) software were used to filter alignment duplications (Li et al., 2009; Freed et al., 2017). GATK (version 4.1.4.0) and vcftools (version 4.2) were used for SNP identification and filtration (parameters: MQ < 50.0 || QD < 2.0, -min-alleles 2 -max-alleles 2 -maf 0.05 -max-missing 0.9, and -cluster-size 3 -cluster-window size 10) (McKenna et al., 2010; Danecek et al., 2011). At last, a total of 1,707,194 informative SNPs were acquired, and the original SNPs were obtained from published data of our lab (Tang, 2019; Ding et al., 2020).

### Association study of plant height

To analyze the natural population structure and linkage disequilibrium (LD) decay, ADMIXTURE (Version 1.3.0) (Alexander et al., 2009), Q+K model, and PopLDdecay (Zhang et al., 2018) were performed according to detailed descriptions from previous studies (Zhao et al., 2022). Three software and models were used, including the general linear model (GLM) in trait analysis by association, evolution, and linkage (TASSEL, Version 5.0) (http://www.maizegenetics.net/tassel); mixed linear model (MLM) in Efficient Mixed-Model Association eXpedited (EMMAX); and Bayesian information and Linkage-disequilibrium Iteratively Nested Keyway (BLINK) (Huang et al., 2019). TASSEL was used to calculate the kinship of 230 *B. napus* accessions (Yu et al., 2006). The LD block was displayed using LDBlockShow software (Dong et al., 2020).

### Subcellular localization

Complete coding sequence of *BnaA01.GSK3* was amplified from the *B. napus* cv. Zhongshuang11 (ZS11). The purified DNA fragment was fused with green fluorescent protein (GFP) in the backbone vector pBWA(V)HS-gfp, resulting in the plasmid *35S:BnaA01.BIN2-GFP via* the ClonExpressMultiS One Step Cloning Kit C113-01 (Vazyme). The *35S:GFP* plasmid was used as the mock control. These plasmids were transiently transformed into *Arabidopsis* protoplast cells using the Agrobacterium-mediated method. The subcellular localization of BnaA01.BIN2 was determined by observing GFP using a Nikon C2-ER confocal microscope (Nikon, Japan) (Zhao et al., 2021). The primers used for amplification of *BnaA01.BIN2* are listed in **Supplementary Table 4**.

### Identification and distribution, structure and conserved domain analysis of *BnGSK3s* family

The amino acid sequences of *AtGSK3s* family were obtained from the database “The Arabidopsis Information Resource (TAIR; https://www.arabidopsis.org/),” which were used to build a Hidden Markov Model, and HMMER3.0 was used to search the annotation and genome information of *B. napus* “*Darmor-bzh*” in the *Brassicaceae* Database (BRAD) (Mistry et al., 2013; Chalhoub et al., 2014; Chen et al., 2021a). The isoelectric point (pI) and molecular weight (MW) of BnGSK3s proteins were predicted using ProtParam online software (https://web.expasy.org/protparam/).

The National Center for Biotechnology Information (NCBI) Conserved Domain Database (https://www.ncbi.nlm.nih.gov/Structure/cdd/wrpsb.cgi) and the SMART database (http://smart.embl.de/) were performed to verify the candidate *BnGSK3s* (Lu et al., 2020; Letunic et al., 2021). Chromosomal locations of the candidate *BnGSK3s* were visualized *via* MapGene2Chromosome V2 (MG2C, http://mg2c.iask.in/mg2c_v2.0/).

The sequences of *BnaGSK3s* were downloaded from BRAD and the gene structures were displayed by Tbtools. The conserved motifs were analyzed by Multiple Expectation Maximization for Motif Elicitation (MEME, http://meme-suite.org) (Bailey et al., 2015; Chen et al., 2020).

### Phylogenetic and syntenic analysis of BnGSK3s

The alignment of the amino acid sequences of AtGSK3s and BnGSK3s was performed by ClustalW (Larkin et al., 2007). Phylogenetic tree was constructed and visualized using the neighbor-joining (NJ) method in MEGA11 software with 1,000 bootstrap replications (Tamura et al., 2021), and visualized by Evolview7 software (He et al., 2016). The syntenic analysis of *GSK3s* between *AtGSK3s* and *BnGSK3s* were obtained from the BRAD database.

### RNA-seq, synthesis of cDNA, and quantitative real-time PCR analysis

The RNA-seq data generated from 13 tissues of ZS11, including roots, SAM, stems, leaves, buds, siliques, stamens, pistils, blossomy petals, wilting petals, sepals, ovules, and pericarps, was previously published in our lab (Sequence Read Archive accession: PRJNA474576 in NCBI and CNP0001630 in China National GeneBank DataBase) were used for the expression pattern analysis of the *BnGSK3s* family (Li et al., 2019b; Dong et al., 2021). FastPure Plant Total RNA Isolation Kit RC401 (Vazyme) was used to extracted total RNA from three biological replicates of different tissues of ZS11, including the roots, SAM, stems, leaves, buds, and siliques using the. First-strand cDNA was generated using a HiScript III 1^st^ Strand cDNA Synthesis Kit (+gDNA wiper) R312 (Vazyme). Quantitative real-time PCR (qRT-PCR) was performed according to a previously described protocol, and the *BnActin* gene was used as an internal control to quantify the relative expression levels of target genes (Zhao et al., 2021). Gene-specific primers for *BnGSK3s* used for qRT-PCR were obtained from the qPrimerDB qPCR Primer Database (Lu et al., 2018) and the corresponding sequences were listed in **Supplementary Table 4**. The heatmap is illustrated using OmicShare Tools (https://www.omicshare.com/tools/).

## Data availability statement

Publicly available datasets were analyzed in this study. This data can be found here: https://bnaomics.ocri-genomics.net/tools/jb-dev/?data=data%2FBna_darmor_v4.1.

## Author contributions

CZ, MX, and XC designed this study and provided the funding. SL supervised the study. CZ and MX performed experiments and wrote the manuscript. LY, XC, MT, YX, and LL provided the plant materials and collected the data. LY assisted in data analysis. LY and MX revised the manuscript. All authors contributed to the article and approved the submitted version.

## Funding

This work was supported by the National Natural Science Foundation of China (32101813, 32070217), Central Public-interest Scientific Institution Basal Research Fund (CAAS-OCRI-XKPY-202104), China Agriculture Research System of MOF and MARA (CARS-12), and the Agricultural Science and Technology Innovation Program of the Chinese Academy of Agricultural Sciences (CAAS-ASTIP-2013-OCRI). Precursor projects of Guizhou province for biological breeding supporting by science and technology in 2022 (Fine identification and evaluation of crop germplasm resources). LY was supported by China Scholarship Council (201903250085).

## Conflict of interest

The authors declare that the research was conducted in the absence of any commercial or financial relationships that could be construed as a potential conflict of interest.

## Publisher’s note

All claims expressed in this article are solely those of the authors and do not necessarily represent those of their affiliated organizations, or those of the publisher, the editors and the reviewers. Any product that may be evaluated in this article, or claim that may be made by its manufacturer, is not guaranteed or endorsed by the publisher.

## Supplementary material

Supplementary Figure 1 Phenotype distribution and correlation of plant height in two consecutive years in *B. napus*. (A) Phenotype distribution of plant height in 2017-2018, 2018-2019, and BLUP. (B) Correlation of plant height between 2017-2018 and 2018-2019.

Supplementary Figure 2 Population structure of 230 *B. napus* accessions. (A) Cross−validation error under different K values. (B) Model-based population structure under K = 9. The y axis represents clusters memberships and the x axis represents the 230 *B. napus* accessions. (C) Relative kinship of 230 rapeseed accessions.

Supplementary Figure 3 Quantile-quantile plots of GWAS in two consecutive years under three models.

## References

[B1] Alexander D. H. Novembre J. Lange K. (2009). Fast model-based estimation of ancestry in unrelated individuals. Genome Res. 19, 1655–1664. doi: 10.1101/gr.094052.109 19648217 PMC2752134

[B2] Anne P. Azzopardi M. Gissot L. Beaubiat S. Hématy K. Palauqui J. C. (2015). OCTOPUS negatively regulates BIN2 to control phloem differentiation in *Arabidopsis thaliana* . Curr. Biol. 25, 2584–2590. doi: 10.1016/j.cub.2015.08.033 26387715

[B3] Bailey T. L. Johnson J. Grant C. E. Noble W. S. (2015). The MEME suite. Nucleic Acids Res. 43, W39–W49. doi: 10.1093/nar/gkv416 25953851 PMC4489269

[B4] Buerstmayr M. Lemmens M. Steiner B. Buerstmayr H. (2011). Advanced backcross QTL mapping of resistance to fusarium head blight and plant morphological traits in a triticum macha x t. aestivum population. Theor. Appl. Genet. 123, 293–306. doi: 10.1007/s00122-011-1584-x 21479934 PMC3114081

[B5] Cai D. Xiao Y. Yang W. Ye W. Wang B. Younas M.. (2014). Association mapping of six yield-related traits in rapeseed (*Brassica napus* l.). Theor. Appl. Genet. 127, 85–96. doi: 10.1007/s00122-013-2203-9 24121524

[B6] Chai L. Xin M. Dong C. Chen Z. Zhai H. Zhuang J.. (2022). A natural variation in ribonuclease h-like gene underlies *Rht8* to confer “Green revolution” trait in wheat. *Mol* . Plant 15, 377–380. doi: 10.1016/j.molp.2022.01.013 35063659

[B7] Chalhoub B. Denoeud F. Liu S. Parkin I. A. P. Tang H. Wang X.. (2014). Early allopolyploid evolution in the post-neolithic *Brassica napus* oilseed genome. Science 345, 950–953. doi: 10.1126/science.1253435 25146293

[B8] Chen C. Chen H. Zhang Y. Thomas H. R. Frank M. H. He Y.. (2020). TBtools: An integrative toolkit developed for interactive analyses of big biological data. Mol. Plant 13, 1194–1202. doi: 10.1016/j.molp.2020.06.009 32585190

[B9] Cheng Y. Zhu W. Chen Y. Ito S. Asami T. Wang X. (2014). Brassinosteroids control root epidermal cell fate *via* direct regulation of a MYB-bHLH-WD40 complex by GSK3-like kinases. Elife 3, e02525. doi: 10.7554/eLife.02525.022 PMC4005458 24771765

[B10] Chen H. Wang T. He X. Cai X. Lin R. Liang J.. (2021a). BRAD V3.0: an upgraded brassicaceae database. Nucleic Acids Res. 50, D1432–D1441. doi: 10.1093/nar/gkab1057 PMC8728314 34755871

[B11] Chen L. Yang H. Fang Y. Guo W. Chen H. Zhang X.. (2021b). Overexpression of GmMYB14 improves high-density yield and drought tolerance of soybean through regulating plant architecture mediated by the brassinosteroid pathway. Plant Biotechnol. J. 19, 702–716. doi: 10.1111/pbi.13496 33098207 PMC8051608

[B12] Cho H. Ryu H. Rho S. Hill K. Smith S. Audenaert D.. (2014). A secreted peptide acts on BIN2-mediated phosphorylation of ARFs to potentiate auxin response during lateral root development. Nat. Cell Biol. 16, 66–76. doi: 10.1038/ncb2893 24362628

[B13] Chu C. G. Xu S. S. Friesen T. L. Faris J. D. (2008). Whole genome mapping in a wheat doubled haploid population using SSRs and TRAPs and the identification of QTL for agronomic traits. Mol. Breed. 22, 251–266. doi: 10.1007/s11032-008-9171-9

[B14] Danecek P. Auton A. Abecasis G. Albers C. A. Banks E. Depristo M. A.. (2011). The variant call format and VCFtools. Bioinformatics 27, 2156–2158. doi: 10.1093/bioinformatics/btr330 21653522 PMC3137218

[B15] Ding L. N. Li M. Guo X. J. Tang M. Q. Cao J. Wang Z.. (2020). *Arabidopsis* GDSL1 overexpression enhances rapeseed sclerotinia sclerotiorum resistance and the functional identification of its homolog in *Brassica napus* . Plant Biotechnol. J. 18, 1255–1270. doi: 10.1111/pbi.13289 31693306 PMC7152613

[B16] Dong Z. Alam M. K. Xie M. Yang L. Liu J. Helal M. M. U.. (2021). Mapping of a major QTL controlling plant height using a high-density genetic map and QTL-seq methods based on whole-genome resequencing in *Brassica napus* . G3-GENES Genom. Genet. 11, jkab118. doi: 10.1093/g3journal/jkab118 PMC8495924 33836054

[B17] Dong S.-S. He W.-M. Ji J.-J. Zhang C. Guo Y. Yang T.-L. (2020). LDBlockShow: a fast and convenient tool for visualizing linkage disequilibrium and haplotype blocks based on variant call format files. Brief. Bioinform. 22, bbaa227. doi: 10.1093/bib/bbaa227 33126247

[B18] Fang Z. Ji Y. Hu J. Guo R. Sun S. Wang X. (2020). Strigolactones and brassinosteroids antagonistically regulate the stability of the D53-OsBZR1 complex to determine FC1 expression in rice tillering. Mol. Plant 13, 586–597. doi: 10.1016/j.molp.2019.12.005 31837469

[B19] Fang N. Xu R. Huang L. Zhang B. Duan P. Li N.. (2016). SMALL GRAIN 11 controls grain size, grain number and grain yield in rice. Rice 9, 64. doi: 10.1186/s12284-016-0136-z 27900723 PMC5127926

[B20] Ferrero-Serrano Á. Su Z. Assmann S. M. (2018). Illuminating the role of the gα heterotrimeric G protein subunit, RGA1, in regulating photoprotection and photoavoidance in rice. Plant Cell Environ. 41, 451–468. doi: 10.1111/pce.13113 29216416

[B21] Freed D. Aldana R. Weber J. A. Edwards J. S. (2017). The sentieon genomics tools - a fast and accurate solution to variant calling from next-generation sequence data. BioRxiv 115717. doi: 10.1101/115717

[B22] Guo W. Chen L. Herrera-Estrella L. Cao D. Tran L.-S. P. (2020). Altering plant architecture to improve performance and resistance. Trends Plant Sci. 25, 1154–1170. doi: 10.1016/j.tplants.2020.05.009 32595089

[B23] Guo Z. Liu X. Zhang B. Yuan X. Xing Y. Liu H.. (2021). Genetic analyses of lodging resistance and yield provide insights into post-Green-Revolution breeding in rice. Plant Biotechnol. J. 19, 814–829. doi: 10.1111/pbi.13509 33159401 PMC8051602

[B24] Guo J. Shi W. Zhang Z. Cheng J. Sun D. Yu J.. (2018). Association of yield-related traits in founder genotypes and derivatives of common wheat (*Triticum aestivum* l.). BMC Plant Biol. 18, 38. doi: 10.1186/s12870-018-1234-4 29458339 PMC5819277

[B25] Hedden P. (2003). The genes of the green revolution. Trends Genet. 19, 5–9. doi: 10.1016/S0168-9525(02)00009-4 12493241

[B26] He Z. Zhang H. Gao S. Lercher M. J. Chen W. H. Hu S. (2016). Evolview v2: an online visualization and management tool for customized and annotated phylogenetic trees. Nucleic Acids Res. 44, W236–W241. doi: 10.1093/nar/gkw370 27131786 PMC4987921

[B27] Hirano K. Asano K. Tsuji H. Kawamura M. Mori H. Kitano H.. (2010). Characterization of the molecular mechanism underlying gibberellin perception complex formation in rice. Plant Cell 22, 2680–2696. doi: 10.1105/tpc.110.075549 20716699 PMC2947161

[B28] Huang M. Liu X. Zhou Y. Summers R. M. Zhang Z. (2019). BLINK: a package for the next level of genome-wide association studies with both individuals and markers in the millions. Gigascience 8, giy154. doi: 10.1093/gigascience/giy154 PMC6365300 30535326

[B29] Islam N. Evans E. J. (1994). Influence of lodging and nitrogen rate on the yield and yield attributes of oilseed rape (*Brassica napus* l.). Theor. Appl. Genet. 88, 530–534. doi: 10.1007/BF01240914 24186106

[B30] Jiang H. Tang B. Xie Z. Nolan T. Ye H. Song G. Y.. (2019). GSK3-like kinase BIN2 phosphorylates RD26 to potentiate drought signaling in *Arabidopsis* . Plant J. 100, 923–937. doi: 10.1111/tpj.14484 31357236

[B31] Jiao Y. Wang Y. Xue D. Wang J. Yan M. Liu G.. (2010). Regulation of OsSPL14 by OsmiR156 defines ideal plant architecture in rice. Nat. Genet. 42, 541–544. doi: 10.1038/ng.591 20495565

[B32] Larkin M. A. Blackshields G. Brown N. P. Chenna R. Mcgettigan P. A. Mcwilliam H.. (2007). Clustal W and clustal X version 2.0. Bioinformatics 23, 2947–2948. doi: 10.1093/bioinformatics/btm404 17846036

[B33] Letunic I. Khedkar S. Bork P. (2021). SMART: recent updates, new developments and status in 2020. Nucleic. Acids Res. 49, D458–D460. doi: 10.1093/nar/gkaa937 33104802 PMC7778883

[B34] Li Y. Dong C. Hu M. Bai Z. Tong C. Zuo R.. (2019b). Identification of flower-specific promoters through comparative transcriptome analysis in *Brassica napus* . Int. J. Mol. Sci. 20, 5949. doi: 10.3390/ijms20235949 PMC6928827 31779216

[B35] Li H. Durbin R. (2009). Fast and accurate short read alignment with burrows-wheeler transform. Bioinformatics 25, 1754–1760. doi: 10.1093/bioinformatics/btp324 19451168 PMC2705234

[B36] Li H. Handsaker B. Wysoker A. Fennell T. Ruan J. Homer N.. (2009). The sequence Alignment/Map format and SAMtools. Bioinformatics 25, 2078–2079. doi: 10.1093/bioinformatics/btp352 19505943 PMC2723002

[B37] Li H. Li J. Song J. Zhao B. Guo C. Wang B.. (2019a). An auxin signaling gene BnaA3.IAA7 contributes to improved plant architecture and yield heterosis in rapeseed. New Phytol. 222, 837–851. doi: 10.1111/nph.15632 30536633

[B38] Li J. Terzaghi W. Gong Y. Li C. Ling J. J. Fan Y.. (2020b). Modulation of BIN2 kinase activity by HY5 controls hypocotyl elongation in the light. Nat. Commun. 11, 1592. doi: 10.1038/s41467-020-15394-7 32221308 PMC7101348

[B39] Liu S. Raman H. Xiang Y. Zhao C. Huang J. Zhang Y. (2022). *De novo* design of future rapeseed crops: Challenges and opportunities. Crop J. 10, 587–596. doi: 10.1016/j.cj.2022.05.003

[B40] Liu C. Wang J. Huang T. Wang F. Yuan F. Cheng X.. (2010). A missense mutation in the VHYNP motif of a DELLA protein causes a semi-dwarf mutant phenotype in *Brassica napus* . Theor. Appl. Genet. 121, 249–258. doi: 10.1007/s00122-010-1306-9 20221582

[B41] Liu S. Zhang M. Feng F. Tian Z. (2020). Toward a “Green revolution” for soybean. Mol. Plant 13, 688–697. doi: 10.1016/j.molp.2020.03.002 32171732

[B42] Li H. Wang L. Liu M. Dong Z. Li Q. Fei S.. (2020a). Maize plant architecture is regulated by the ethylene biosynthetic gene ZmACS7. Plant Physiol. 183, 1184–1199. doi: 10.1104/pp.19.01421 32321843 PMC7333711

[B43] Lu K. Li T. He J. Chang W. Zhang R. Liu M.. (2018). qPrimerDB: a thermodynamics-based gene-specific qPCR primer database for 147 organisms. Nucleic Acids Res. 46, D1229–D1236. doi: 10.1093/nar/gkx725 28977518 PMC5753361

[B44] Lu S. Wang J. Chitsaz F. Derbyshire M. K. Geer R. C. Gonzales N. R.. (2020). CDD/SPARCLE: the conserved domain database in 2020. Nucleic Acids Res. 48, D265–D268. doi: 10.1093/nar/gkz991 31777944 PMC6943070

[B45] Mao J. Li J. (2020). Regulation of three key kinases of brassinosteroid signaling pathway. Int. J. Mol. Sci. 21, 4340. doi: 10.3390/ijms21124340 PMC7352359 32570783

[B46] McKenna A. Hanna M. Banks E. Sivachenko A. Cibulskis K. Kernytsky A.. (2010). The genome analysis toolkit: a MapReduce framework for analyzing next-generation DNA sequencing data. Genome Res. 20, 1297–1303. doi: 10.1101/gr.107524.110 20644199 PMC2928508

[B47] Mistry J. Finn R. D. Eddy S. R. Bateman A. Punta M. (2013). Challenges in homology search: HMMER3 and convergent evolution of coiled-coil regions. Nucleic Acids Res. 41, e121. doi: 10.1093/nar/gkt263 23598997 PMC3695513

[B48] Miura K. Ikeda M. Matsubara A. Song X. J. Ito M. Asano K.. (2010). OsSPL14 promotes panicle branching and higher grain productivity in rice. Nat. Genet. 42, 545–549. doi: 10.1038/ng.592 20495564

[B49] Monna L. Kitazawa N. Yoshino R. Suzuki J. Masuda H. Maehara Y.. (2002). Positional cloning of rice semidwarfing gene, sd-1: Rice “Green revolution gene” encodes a mutant enzyme involved in gibberellin synthesis. DNA Res. 9, 11. doi: 10.1093/dnares/9.1.11 11939564

[B50] Pearce S. (2021). Towards the replacement of wheat ‘Green revolution’ genes. J. Exp. Bot. 72, 157–160. doi: 10.1093/jxb/eraa494 33529341 PMC7853296

[B51] Peng J. Richards D. E. Hartley N. M. Murphy G. P. Devos K. M. Flintham J. E.. (1999). ‘Green revolution’ genes encode mutant gibberellin response modulators. Nature 400, 256. doi: 10.1038/22307 10421366

[B52] Phillips K. A. Skirpan A. L. Liu X. Christensen A. Slewinski T. L. Hudson C.. (2011). Vanishing tassel2 encodes a grass-specific tryptophan aminotransferase required for vegetative and reproductive development in maize. Plant Cell 23, 550–566. doi: 10.1105/tpc.110.075267 21335375 PMC3077783

[B53] Sasaki A. Ashikari M. Ueguchitanaka M. Itoh H. Nishimura A. Swapan D.. (2002). Green revolution: a mutant gibberellin-synthesis gene in rice. Nature 416, 701–702. doi: 10.1038/416701a 11961544

[B54] Spielmeyer W. Ellis M. H. Chandler P. M. (2002). Semidwarf (sd-1), “green revolution” rice, contains a defective gibberellin 20-oxidase gene. P. Natl. Acad. Sci. U.S.A. 99, 9043–9048. doi: 10.1073/pnas.132266399 PMC124420 12077303

[B55] Sun S. Wang L. Mao H. Shao L. Li X. Xiao J.. (2018). A G-protein pathway determines grain size in rice. Nat. Commun. 9, 851. doi: 10.1038/s41467-018-03141-y 29487318 PMC5829277

[B56] Sun C. Wang B. Yan L. Hu K. Liu S. Zhou Y.. (2016). Genome-wide association study provides insight into the genetic control of plant height in rapeseed (*Brassica napus* l.). Front. Plant Sci. 7, 1102. doi: 10.3389/fpls.2016.01102 27512396 PMC4961929

[B57] Tamura K. Stecher G. Kumar S. (2021). MEGA11: Molecular evolutionary genetics analysis version 11. Mol. Biol. Evol. 38, 3022–3027. doi: 10.1093/molbev/msab120 33892491 PMC8233496

[B58] Tang M. Q. (2019). Population genome variations and subgenome asymmetry in brassica napus l (Wuhan, China: Huazhong Agricultural University), D50–D59.

[B59] Tan Z. Xie Z. Dai L. Zhang Y. Zhao H. Tang S.. (2022). Genome- and transcriptome-wide association studies reveal the genetic basis and the breeding history of seed glucosinolate content in *Brassica napus* . Plant Biotechnol. J. 20, 211–225. doi: 10.1111/pbi.13707 34525252 PMC8710833

[B60] Teichmann T. Muhr M. (2015). Shaping plant architecture. Front. Plant Sci. 6, 233. doi: 10.3389/fpls.2015.00233 25914710 PMC4390985

[B61] Wang Y. Chen W. Chu P. Wan S. Yang M. Wang M.. (2016a). Mapping a major QTL responsible for dwarf architecture in *Brassica napus* using a single-nucleotide polymorphism marker approach. BMC Plant Biol. 16, 178. doi: 10.1186/s12870-016-0865-6 27538713 PMC4991092

[B62] Wang Y. He J. Yang L. Wang Y. Chen W. Wan S.. (2016b). Fine mapping of a major locus controlling plant height using a high-density single-nucleotide polymorphism map in brassica napus. Theor. Appl. Genet. 129, 1479–1491. doi: 10.1007/s00122-016-2718-y 27147069

[B63] Wang Y. Li J. (2008). Molecular basis of plant architecture. Annu. Rev. Plant Biol. 59, 253–279. doi: 10.1146/annurev.arplant.59.032607.092902 18444901

[B64] Wang Y. Shang L. Yu H. Zeng L. Hu J. Ni S.. (2020b). A strigolactone biosynthesis gene contributed to the green revolution in rice. Mol. Plant 13, 923–932. doi: 10.1016/j.molp.2020.03.009 32222483

[B65] Wang H. Tang J. Liu J. Hu J. Liu J. Chen Y.. (2018). Abscisic acid signaling inhibits brassinosteroid signaling through dampening the dephosphorylation of BIN2 by ABI1 and ABI2. Mol. Plant 11, 315–325. doi: 10.1016/j.molp.2017.12.013 29275167

[B66] Wang X. Zheng M. Liu H. Zhang L. Chen F. Zhang W.. (2020a). Fine-mapping and transcriptome analysis of a candidate gene controlling plant height in *Brassica napus* l. Biotechnol. Biofuels. 13, 42. doi: 10.1186/s13068-020-01687-y 32175009 PMC7063735

[B67] Xiong H. Zhou C. Fu M. Guo H. Xie Y. Zhao L.. (2022). Cloning and functional characterization of *Rht8*, a “Green revolution” replacement gene in wheat. Mol. Plant 15, 373–376. doi: 10.1016/j.molp.2022.01.014 35063661

[B68] Xu J. Ni Z. Chen F. Fu X. Yu F. (2022). Integrated linkage mapping and genome-wide association study to dissect the genetic basis of zinc deficiency tolerance in maize at seedling stage. Crop J. doi: 10.1016/j.cj.2022.05.004

[B69] Yang M. He J. Wan S. Li W. Chen W. Wang Y.. (2021). Fine mapping of the *BnaC04.BIL1* gene controlling plant height in *Brassica napus* L. BMC Plant Biol. 21, 359. doi: 10.1186/s12870-021-03137-9 34353289 PMC8340546

[B70] Ye H. Feng J. Zhang L. Zhang J. Mispan M. S. Cao Z.. (2015). Map-based cloning of seed Dormancy1-2 identified a gibberellin synthesis gene regulating the development of endosperm-imposed dormancy in rice. Plant Physiol. 169, 2152–2165. doi: 10.1104/pp.15.01202 26373662 PMC4634095

[B71] Yu J. Pressoir G. Briggs W. H. Vroh Bi I. Yamasaki M. Doebley J. F.. (2006). A unified mixed-model method for association mapping that accounts for multiple levels of relatedness. Nat. Genet. 38, 203–208. doi: 10.1038/ng1702 16380716

[B72] Zhang C. Dong S.-S. Xu J.-Y. He W.-M. Yang T.-L. (2018). PopLDdecay: a fast and effective tool for linkage disequilibrium decay analysis based on variant call format files. Bioinformatics 35, 1786–1788. doi: 10.1093/bioinformatics/bty875 30321304

[B73] Zhao B. Li H. Li J. Wang B. Dai C. Wang J.. (2017). Brassica napus DS-3, encoding a DELLA protein, negatively regulates stem elongation through gibberellin signaling pathway. Theor. Appl. Genet. 130, 727–741. doi: 10.1007/s00122-016-2846-4 28093630

[B74] Zhao C. Safdar L. B. Xie M. Shi M. Dong Z. Yang L.. (2021). Mutation of the PHYTOENE DESATURASE 3 gene causes yellowish-white petals in *Brassica napus* . Crop J. 9, 1124–1134. doi: 10.1016/j.cj.2020.10.012

[B75] Zhao B. Wang B. Li Z. Guo T. Zhao J. Guan Z.. (2019). Identification and characterization of a new dwarf locus DS-4 encoding an Aux/IAA7 protein in brassica napus. Theor. Appl. Genet. 132, 1435–1449. doi: 10.1007/s00122-019-03290-8 30688990

[B76] Zhao C. Xie M. Liang L. Yang L. Han H. Qin X.. (2022). Genome-wide association analysis combined with quantitative trait loci mapping and dynamic transcriptome unveil the genetic control of seed oil content in *Brassica napus* l. Front. Plant Sci. 13, 929197. doi: 10.3389/fpls.2022.929197 35845656 PMC9283957

[B77] Zheng M. Hu M. Yang H. Tang M. Zhang L. Liu H.. (2019). Three BnaIAA7 homologs are involved in auxin/brassinosteroid-mediated plant morphogenesis in rapeseed (*Brassica napus* l.). Plant Cell Rep. 38, 883–897. doi: 10.1007/s00299-019-02410-4 31011789 PMC6647246

[B78] Zheng M. Terzaghi W. Wang H. Hua W. (2022). Integrated strategies for increasing rapeseed yield. Trends Plant Sci. 27, 742–745. doi: 10.1016/j.tplants.2022.03.008 35501261

