# Supplementary figures and images for: Genome-wide association study reveals a GLYCOGEN SYNTHASE KINASE 3 gene regulating plant height in Brassica napus
Source: Front Plant Sci. 2022 Nov 2;13:1061196. doi: 10.3389/fpls.2022.1061196 (PMC9666772; doi:10.3389/fpls.2022.1061196)

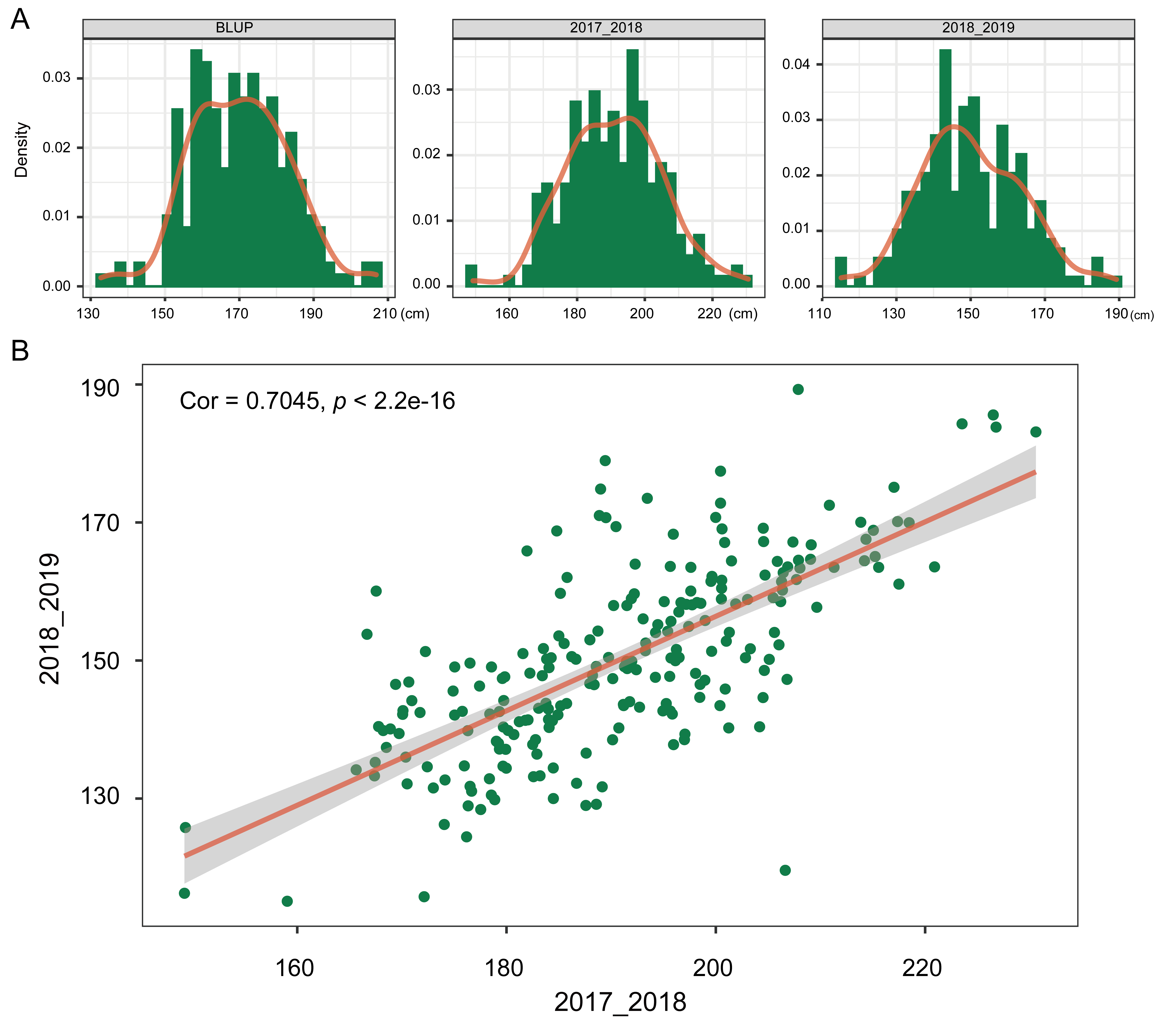

Supplement: Supplementary Figure 1 — Phenotype distribution and correlation of plant height in two consecutive years in B. napus. (A) Phenotype distribution of plant height in 2017-2018, 2018-2019, and BLUP. (B) Correlation of plant height between 2017-2018 and 2018-2019. [file Image_1.tif]

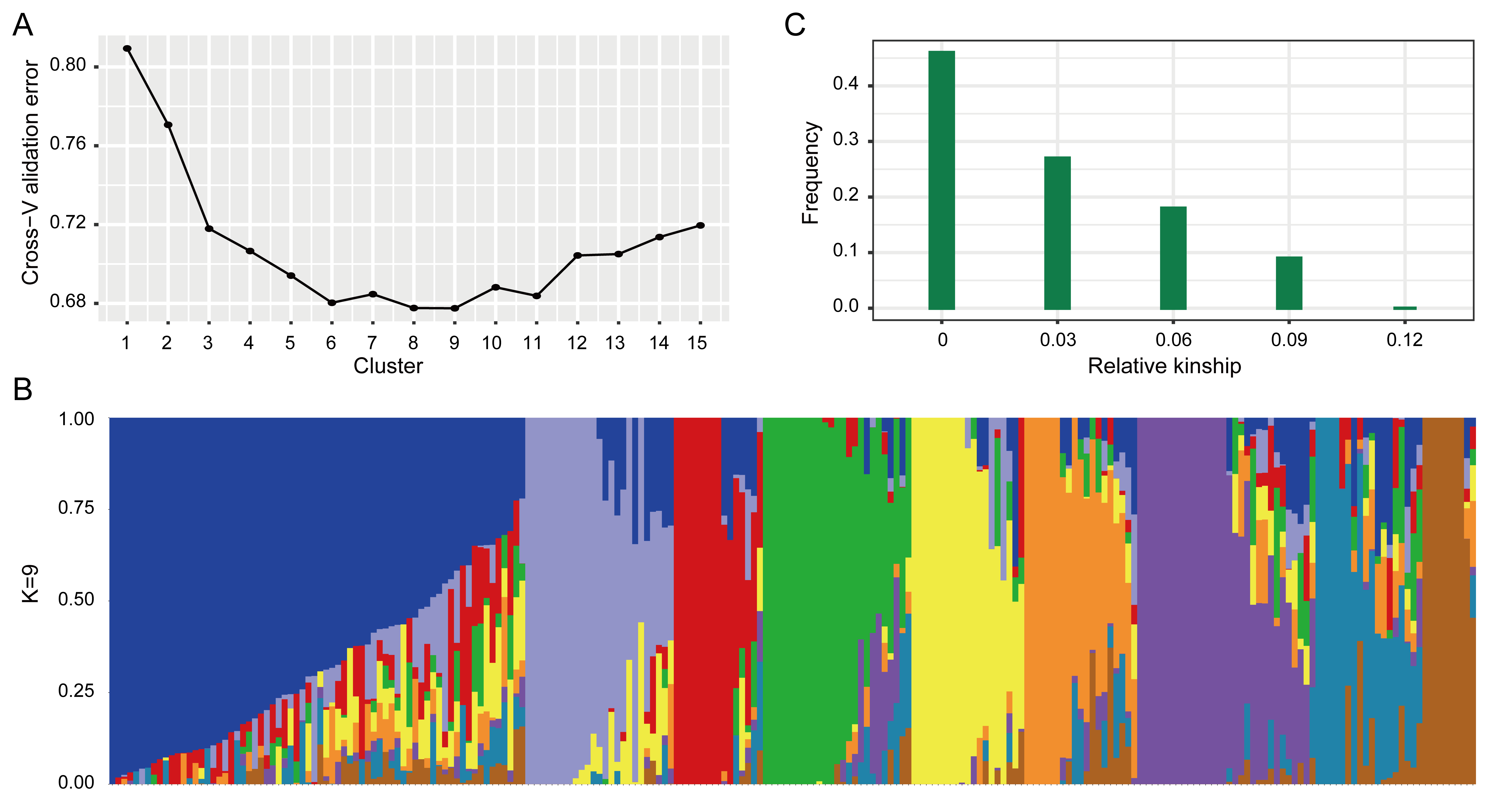

Supplement: Supplementary Figure 2 — Population structure of 230 B. napus accessions. (A) Cross−validation error under different K values. (B) Model-based population structure under K = 9. The y axis represents clusters memberships and the x axis represents the 230 B. napus accessions. (C) Relative kinship of 230 rapeseed accessions. [file Image_2.tif]
